# Routine Clinical Measures of Adiposity as Predictors of Visceral Fat in Adolescence: A Population-Based Magnetic Resonance Imaging Study

**DOI:** 10.1371/journal.pone.0079896

**Published:** 2013-11-11

**Authors:** Katie Goodwin, Catriona Syme, Michal Abrahamowicz, Gabriel T. Leonard, Louis Richer, Michel Perron, Suzanne Veillette, Daniel Gaudet, Tomas Paus, Zdenka Pausova

**Affiliations:** 1 Hospital for Sick Children, University of Toronto, Toronto, Canada; 2 Department of Epidemiology, Biostatistics and Occupational Health, McGill University, Montreal, Canada; 3 Montreal Neurological Institute, McGill University, Montreal, Canada; 4 Department of Psychology, Université du Québec à Chicoutimi, Chicoutimi, Canada; 5 Department of Human Sciences, Université du Québec à Chicoutimi, Chicoutimi, Canada; 6 Community Genomic Centre, Université de Montréal, Chicoutimi Hospital, Chicoutimi, Canada; 7 Rotman Research Institute, University of Toronto, Toronto, Canada; Scientific Directorate, Bambino Hospital, Italy

## Abstract

**Objective:**

Visceral fat (VF) increases cardiometabolic risk more than fat stored subcutaneously. Here, we investigated how well routine clinical measures of adiposity, namely body mass index (BMI) and waist circumference (waist), predict VF and subcutaneous fat (SF) in a large population-based sample of adolescents. As body-fat distribution differs between males and females, we performed these analyses separately in each sex.

**Design and Methods:**

VF and SF were measured by magnetic resonance imaging in 1,002 adolescents (482 males, age 12–18 years). Relationships of BMI and waist with VF and SF were tested in multivariable analyses, which adjusted for potentially confounding effects of age and height.

**Results:**

In both males and females, BMI and waist were highly correlated with VF and SF, and explained 55–76% of their total variance. When VF was adjusted for SF, however, BMI and waist explained, respectively, only 0% and 4% of VF variance in males, and 4% and 11% of VF variance in females. In contrast, when SF was adjusted for VF, BMI and waist explained, respectively, 36% and 21% of SF variance in males, and 48% and 23% of SF variance in females. These relationships were similar during early and late puberty.

**Conclusions and Relevance:**

During adolescence, routine clinical measures of adiposity predict well SF but not VF. This holds for both sexes and throughout puberty. Further longitudinal studies are required to assess how well these measures predict changes of VF and SF over time. Given the clinical importance of VF, development of cost-effective imaging techniques and/or robust biomarkers of VF accumulation that would be suitable in everyday clinical practice is warranted.

## Introduction

 Obesity is a major public health problem [1,2]. Due to its currently high prevalence and adverse effects on health [1,2], our life expectancy has been projected to decline for the first time since the Great Depression [3]. This is in part due to obesity increasing significantly risk for cardio-metabolic diseases (CMD), such as cardiovascular disease and type-2 diabetes mellitus [2,4], which in turn are the major causes of morbidity and mortality in the industrialized world. This obesity-related risk for CMD is mainly the consequence of adipose tissue releasing a number of adipocytokines that, once released into the circulation, promote the development of CMD [5]. 

 The relationship between obesity and CMD is not simple, however. About 30% of adult individuals who are classified as “obese” are cardiometabolically healthy and over 20% who are classified as being of “normal weight” are cardiometabolically abnormal [6]. To some extent, this may be an artefact of the measurement method. For over 150 years, obesity has been assessed with body mass index (BMI, weight/height^2^), which is an imprecise, and possibly misleading [7], metric of body fat. This is because BMI (and waist circumference [waist]) are influenced not only by fat mass but also by muscle mass and bone mass, among others [8]. 

 Further, obesity-related risk for CMD increases not only with the quantity but also with a specific distribution of body fat – individuals who store body fat viscerally rather than elsewhere in the body (mostly subcutaneously) are at a greater risk for CMD [9–11]. This relationship is seen not only in adults, but also in children and adolescents [12–16]. Several mechanistic pathways have been proposed to underlie the link between visceral fat (VF) and CMD - VF, as compared with subcutaneous fat (SF), exhibits a more adverse secretory profile and higher lipid turnover [4,17–19]. Further, VF but not SF drains directly to the portal circulation and liver, where it enhances dyslipidemia and insulin resistance, key mediators of the link between obesity and CMD [4,20,21]. Consistent with these differences between VF and SF, it has been demonstrated that surgical removal of VF but not that of SF improves cardio-metabolic health in humans and experimental animals [22–24]. Moreover, a growing body of research suggests that SF may even be cardiometabolically protective in overweight and obesity [14,25,26]. This effect has been related to the so-called “expandability” of SF, which is thought to be a biological property of SF that allows excess body-fat to accumulate preferentially in this fat depot and thus protects the body from the cardiometabolically adverse accumulation of VF [27,28]. 

 Thus, given the biological and clinical differences between VF and SF and hence the importance of quantifying them separately, the aim of the present study was to investigate how well routine clinical measures of adiposity, namely BMI and waist, predict VF and SF measured directly with magnetic resonance imaging (MRI) in a large population-based sample of adolescents (n=1,002). We focused here on adolescence, as it is an understudied period of human development [29] during which the initial stages of CMD may emerge [13,30–34]. 

## Methods

### Adolescent sample

 The study sample consisted of White Caucasian males (n=482) and females (n=520), aged 12 to 18 years, who were recruited via high schools from the Saguenay-Lac St. Jean region of Quebec, Canada, as part of the Saguenay Youth Study (SYS) [35]. The SYS is a population-based cross-sectional study of cardio-metabolic and mental health during adolescence. Assent of the adolescents and written consent of the parents were obtained before data collection, and the Research Ethics Committees of the Chicoutimi Hospital (Chicoutimi, Canada) and the Hospital for Sick Children (Toronto, Canada) approved the study. The current sample of 1,002 adolescents was recruited and tested between November 2003 and February 2012.

### Routine clinical measures of adiposity

 Height (0.1-cm precision), weight (0.1-kg precision), waist (0.1-cm precision), hip circumference (0.1-cm precision), and suprailiac skinfold-thickness (suprailiac skinfold, 1-mm precision) were measured by trained staff using standard operating procedures [35]. Waist was measured at the level of the natural waist, which is the narrowest part of the torso, as seen from the anterior aspect. Hip circumference was measured at the level of the maximum extension of the buttocks. Suprailiac skinfold was measured in the midaxillary line immediately superior to the iliac crest. BMI was calculated as weight (in kg) divided by height (in m) squared. Waist to hip ratio (waist/hip) was calculated as waist (in cm) divided by hip circumference (in cm). 

### Magnetic resonance imaging of abdominal VF and SF

 VF and SF were quantified with MRI from axial, 10-mm thick (with in-plane resolution 1.56 x 1.56 mm^2^), heavily T1-weighted, spin-echo (TR/TE = 200 ms/20 ms) scans, which were taken along the abdomen with a Phillips 1.0-T magnetic resonance scanner. A single slice at the level of the umbilicus was used to quantify VF and SF. The single slice images were smoothed using an adaptive bilateral filter to remove image noise while preserving edge information. A standard region growing algorithm written in MatLab (R2011a, MathWorks, Natick, MA) was used to obtain an initial fat classification map. An iterative refinement procedure corrected false positives and negatives using morphological operators, including hysteresis, thresholding over small neighbourhoods, and median filtering to remove salt-and-pepper noise. The resultant image was manually segmented into SF and VF using Adobe Photoshop (CS5, Adobe Systems Incorporated, San Jose, CA). SF was defined as the area of adipose tissue between the skin and the outer aspect of abdominal musculature. VF was defined as the as the area of adipose tissue within the inner aspect of abdominal musculature and outside of abdominal organs. A histogram counting algorithm written in MatLab (R2011a, MathWorks, Natick, MA) computed the total number of pixels for each type of fat (VF and SF). All 1,002 scans were segmented by a single individual (KG). This segmentation was validated by another individual (CS) in 586 scans (VF: r=0.92, SF: r=0.99). 

### Pubertal development

Puberty stage (1 to 5) was assessed using Puberty Development Scale, which is an 8-item self-report measure of physical development based on Tanner stages [36]. With this tool, separate forms for males and females are used and the 5 puberty stages (i.e., 1. pre-pubertal, 2. beginning pubertal, 3. mid-pubertal, 4. advanced pubertal and 5. post-pubertal) are assessed based on answers to questions regarding pubic hair, growth in stature, menarche for girls and voice changes in males. This tool was validated against physician’s assessment of pubertal development [37] and correlates well with plasma level of sex hormones [38].

### Statistical methods

 Descriptive statistics used to characterize the study population included means and standard deviations. Our main analyses were aimed at estimating the proportion of variance shared between each of two main clinical measures of adiposity, namely BMI and waist, and VF or SF. We ran two sets of multivariable linear regression models, that all adjusted for inter-individual differences in age and height, and used either VF or SF as the continuous dependent variable. In the first sets of analyses, the models that predicted VF did not adjust for SF, and *vice versa*, whereas in the second sets of analyses, they did. The latter sets of analyses were intended to assess if, and to what extent, BMI and waist predict VF even among adolescents with the same SF, and *vice versa*. The same analytical methods were also employed to examine if VF and SF can be predicted by suprailiac skinfold and waist/hip, which are additional anthropometric measures of central adiposity that could be used in clinical setting. Similar to other studies [29], marked sex differences in body-fat distribution were evident in our sample (Table 1). Therefore, we run all our analyses separately in males and females.”

**Table 1:** Basic characteristics and adiposity measures of studied adolescent males and females.

Variables	Males Mean ±SD	Females Mean ±SD	p-value
Number	482	518	
Age (months)	180 ±21	181 ±23	0.18
Height (cm)	167 ±11	160 ±6.7	<0.0001
Puberty stage (1-5)	10/61/184/197/29	3/3/74/296/143	<0.0001
Stage 1	2%	1%	
Stage 2	13%	1%	
Stage 3	38%	14%	
Stage 4	41%	57%	
Stage 5	6%	27%	
Puberty stage (Early/Late)	255/226	83/439	<0.0001
Body mass index			
kg/m^2^	21.7 ±4.3	21.7 ±4.0	0.87
log kg/m^2^	1.33 ±0.08	1.33 ±0.08	0.75
percentile	58.8 ±28.8	56.1 ±27.3	0.14
Waist			
cm	75.1 ±10	71.0 ±8.8	<0.0001
log cm	1.87 ±0.06	1.85 ±0.05	<0.0001
Hip			
cm	88.9 ±10	89.9 ±9.9	0.11
log cm	1.95 ±0.05	1.95 ±0.05	0.08
Suprailiac skinfold			
mm	16.8 ±12	20.6 ±10	<0.0001
log mm	1.13 ±0.28	1.26 ±0.21	<0.0001
Waist/hip			
cm/cm	0.848 ±0.056	0.791 ±0.062	<0.0001
log cm/cm	-0.073 ±0.028	-0.103 ±0.033	<0.0001
Waist/height			
cm/cm	0.440 ±0.084	0.439 ±0.071	0.88
log cm/cm	-0.350 ±0.051	-0.355 ±0.050	0.14
Visceral fat			
cm^2^	230.49 ±224.98	208.78 ±141.99	0.07
log cm^2^	2.22 ±0.34	2.24 ±0.25	0.17
Subcutaneous fat			
cm^2^	1058.57 ±1028.26	1450.07 ±963.33	<0.0001
log cm^2^	2.86 ±0.36	3.08 ±0.27	<0.0001

Unadjusted mean ± standard deviation for raw and log-transformed values of relevant characteristics are shown for the studied males and females. P values indicate statistical significance of differences between males and females evaluated with 2-sided t test.

As secondary analyses, we examined whether pubertal development influences the observed relationships. To this end, we added the two-way interactions of puberty stage (dichotomized as early [stages 1-3] vs. late [stages 4-5] puberty) with each of the tested main independent variables (BMI, waist, suprailiac skinfold and waist/hip). Again, these analyses were carried out in each sex separately.

In preliminary analyses, distributions of all continuous variables were assessed for the normality assumption on which the statistical inference about the linear model estimates relies. The values of variables for which the empirical distribution showed substantial positive skeweness (normality tested with K–S statistics), namely BMI, waist, suprailiac skinfold, waist/hip, VF and SF were all log transformed, using logarithm with base 10, which improved the fit of the multivariable models in the main analyses (data not shown). 

All statistical analyses were performed using JMP software (Release 9, SAS Institute Inc., Cary, NC).

## Results

### Characteristics of studied adolescent males and females

Mean ages of males (n=482) and females (n=520) differed by only 1 month, but, as expected [39], males were at an earlier stage of pubertal development (p<0.0001, Table 1). Males and females had similar BMI (p=0.75) and VF (p=0.17), but males (vs. females) showed *higher* waist (p<0.0001) and waist/hip (p<0.0001), and *lower* suprailiac skinfold (p<0.0001) and SF (p<0.0001, Table 1). 

**Figure 1:**
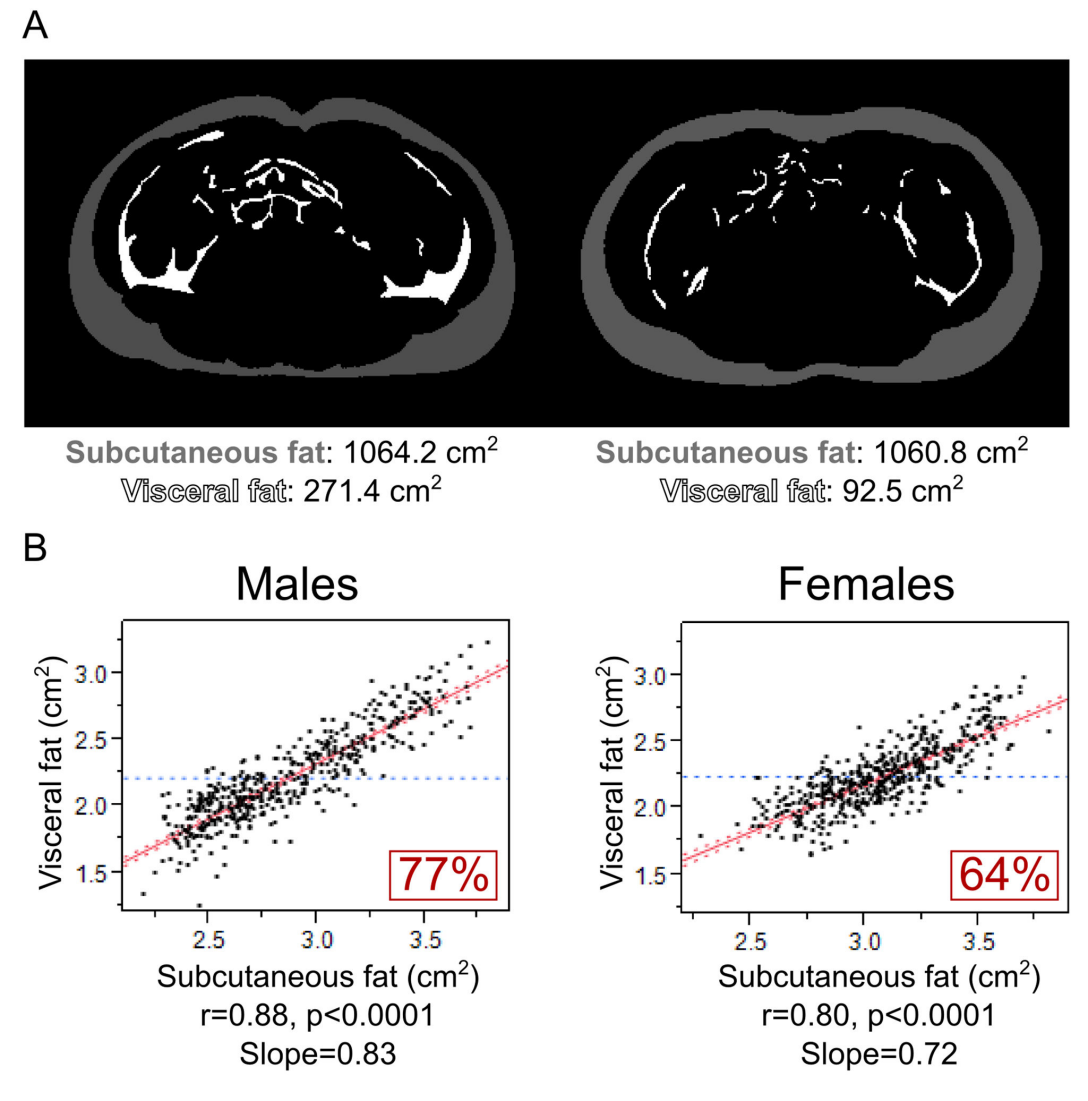
Relationships of VF with SF in adolescent males and females. A) Magnetic resonance images of analyzed umbilical slices in 2 individuals with similar subcutaneous fat and different visceral fat. B) Univariate correlations between of VF with SF are shown in adolescent males and females.

As expected, SF was closely associated with VF; the two measures shared 77% (p<0.0001) and 64% (p<0.0001) of variance in males and females, respectively (Figure 1). The slope of this relationship was steeper in males than females (p=0.04 for the test of interaction with sex) indicating that, for a given quantity of SF, males compared with females have more VF (Figure 1). 

### Routine clinical measures of adiposity as predictors of abdominal VF and SF

 BMI was closely associated with VF and SF (p<0.0001) in both sexes; it explained 58% and 55% of variance of VF and 72% and 76% of variance of SF in males and females of the same age and height, respectively. Relationships of waist with VF and SF were equally strong (p<0.0001); it explained 61% (p<0.0001) and 56% (p<0.0001) of variance of VF and 67% (p<0.0001) and 62% (p<0.0001) of variance of SF in males and females of the same age and height, respectively (Figure S1 in File S1). 

**Figure 2:**
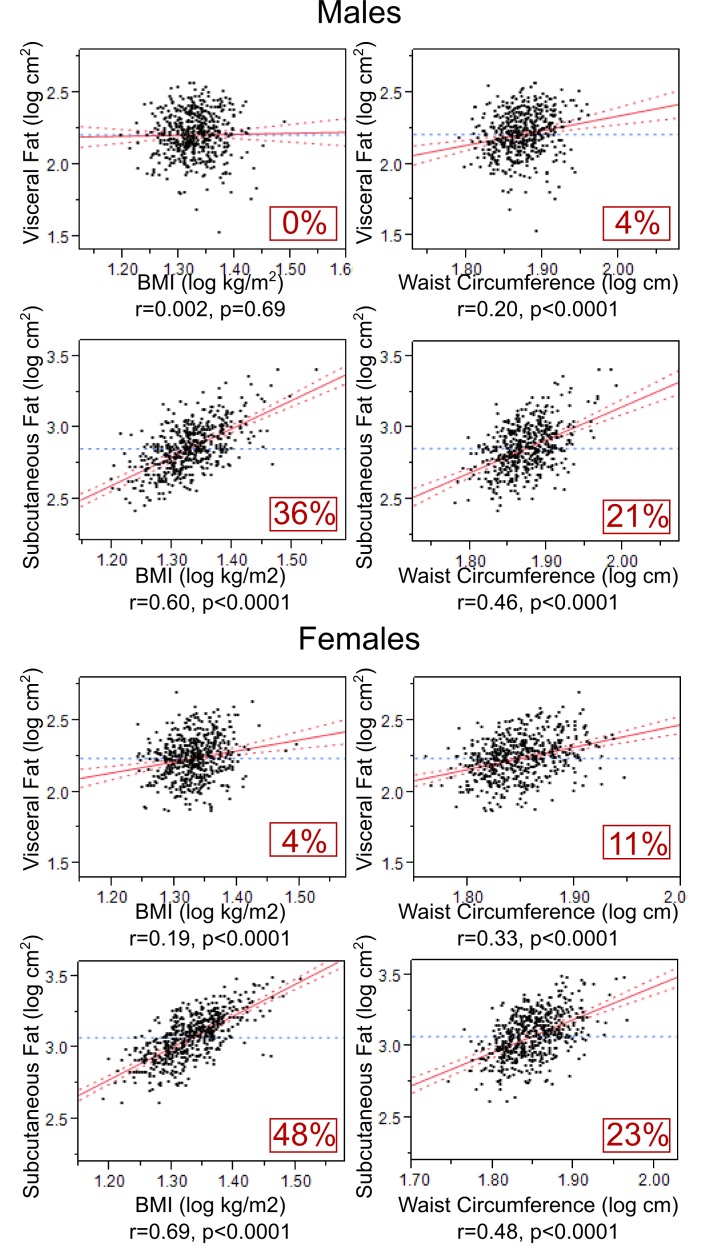
BMI and waist circumference as predictors of VF- and SF-specific quantities (VF and SF adjusted for each other). Multivariate linear regression models examining the relationships of BMI and waist circumference with each VF and SF (while adjusting for each other) are shown in adolescent males and females. All relationships were also adjusted for potentially confounding effects of age and height when appropriate.

 However, in the models predicting VF adjusted for SF, BMI and waist explained, respectively, only 0% (p=0.69) and 4% (p<0.0001) of VF variance (in males); similarly in females, BMI and waist explained, respectively, just 4% (p<0.0001) and 11% (p<0.0001) of VF variance (Figure 2). In contrast, in the models predicting SF adjusted for VF, BMI and waist explained, respectively, substantial proportions of SF variance - 36% (p<0.0001) and 21% (p<0.0001) in males and 48% (p<0.0001) and 23% (p<0.0001) in females (Figure 2). In other words, among both males and females with the same SF, BMI and waist did not relate to VF but, among adolescents with the same VF, these two measures did relate well to SF. 

 Major changes in body size and composition happen during puberty, with accelerated weight gain occurring in most boys and girls at stages 4 and 5 of pubertal development [40]. Therefore, we examined whether the relationships of BMI and waist with VF and SF differ between adolescents who were in early puberty (stages 1-3) vs. those who were in late puberty (stages 4-5, Table S1 in File S1). These analyses showed that relationships of BMI and waist with VF and SF do not substantially differ between the two phases of pubertal development (p>0.30 and p>0.15 for the tests of interaction with puberty stage in males and females, respectively). 

### Additional anthropometric measures as predictors of abdominal VF and SF

 In addition to BMI and waist, we investigated two other anthropometric measures of adiposity that could be used in clinics as predictors of VF and SF, namely suprailiac skinfold and waist/hip. Suprailiac skinfold showed strong relationships with VF and SF in both sexes, explaining 62% (p<0.0001) and 52% (p<0.0001) of VF variance and 72% (p<0.0001) and 64% (p<0.0001) of SF variance in males and females, respectively (Figure S2 in File S1). But again, when VF was adjusted for SF, suprailiac skinfold explained only 2% (p=0.002) and 5% (<0.001) of VF variance in males and females, respectively, and when SF was adjusted for VF, it explained 27% (p<0.0001) and 26% (p<0.0001) of SF variance in males and females, respectively (Figure S3 in File S1). As such, suprailiac skinfold compared with BMI and waist showed a similar potential to predict VF and SF. 

 Waist/hip, however, demonstrated a different pattern of relationships. It showed substantially weaker relationships with both VF or SF (as compared with BMI, waist and suprailiac skinfold ) – it explained only 22% (p<0.0001) and 20% (p<0.0001) of VF variance and 12% (p<0.0001) and 6% (p<0.0001) of SF variance in males and females, respectively (Figure S2 in File S1). Further, when VF was adjusted for SF, waist/hip explained 5% (p<0.0001) and 6% (p<0.0001) of VF variance in males and females, respectively, which was similar to the other anthropometric measures, but unlike them, waist/hip explained 0% of variance in both sexes (p=0.81 and 0.33, respectively) when SF was adjusted for VF (Figure S3 in File S1). As such, waist/hip compared with BMI, waist and suprailiac skinfold showed a similarly low potential to predict VF and an even lower potential to predict SF. 

 The relationships of both these additional anthropometric measures of adiposity (i.e., suprailiac skinfold and waist/hip) with VF and SF did not differ between early and late puberty groups (p>0.29 and p>0.12 for the tests of interaction with puberty stage in males and females, respectively).

## Discussion

 The results of the present study demonstrate that, during adolescence, routine clinical measures of adiposity, namely BMI and waist, predict SF but not VF. These relationships are similar in both sexes and exist throughout puberty. 

 The current study is a large-scale investigation of adolescents (n=1,002) testing BMI and waist as predictors of VF and SF measured directly with MRI. A few such (>500 individuals) investigations have been conducted in adults [41–43] but none in children or adolescents. In the latter age category (children or adolescents), only smaller-scale studies have been performed [44–46]. Irrespective of their size or participants’ age, all of these studies have shown, similarly to the current one (Figure S1 in File S1), that BMI and waist are closely related to both VF and SF and explain large proportions of their respective variances (BMI: 37-69% of VF and 74-89% of SF; waist: 53-70% of VF and 67-86% of SF) [41–46]. But because VF and SF are closely associated with each other (64-77%, Figure 1), it is likely these relationships are driven mainly by global adiposity. Therefore, in the present study (in contrast to previous studies [41–45]), we examined how BMI and waist relate to VF and SF independently of each other – thus, we examined whether BMI and waist can predict VF- and SF-specific quantities of body fat. These results showed that BMI and waist explain a very small proportion of VF-specific variance (0-11%, Figure 2) but a substantially larger proportion of SF-specific variance (21-48%, Figure 2), indicating that both BMI and waist predict mainly SF but not VF.

 In the present study, we examined two additional anthropometric measures of adiposity that could be used in clinics as predictors of VF and SF – these were suprailiac skinfold and waist/hip. Both measures performed either similarly or even worse that BMI and waist (Figures S2 and S3 in File S1).

 The current study has potential limitations. First, it was carried out in White Caucasians and its findings may not be generalizable to other ethnicities. Second, we quantified VF and SF using a single slice. Single-slice data may not be as accurate as multi-slice data in quantifying VF volume [47], but they have been deemed to be a reasonable approximation [48–50]. Third, we quantified VF and SF at the level of the umbilicus. This level may be problematic, as it may correspond to varying locations of the lumbar vertebrae [48,51]. In the present study, we observed that the umbilicus was located within a segment of the body that is 5 to 10 cm above the L4/L5 vertebral disk in most our participants (20 out of 20 randomly selected individuals). VF assessed as a slice area within this segment has been reported as most closely associated with VF volume [48,49], as well as with its age-related increases [52] and risk for CMD [53].

 The results of the present large-scale study suggest that, in adolescence, BMI and waist circumference cannot be used to estimate VF. This poses a challenge for the practice of personalized (preventive) medicine, as youth at risk for obesity-related CMD cannot be easily identified using these clinical measures [54]. Furthermore, the use of BMI and waist circumference may obscure our understanding of obesity-related CMD and thus development of new effective treatments. As described in Introduction, VF vs. SF is more closely associated with obesity-related risk for CMD [9–16]. This is because VF vs. SF is more closely located to the portal circulation [4,20,21] and demonstrates a more adverse secretory profile [4,17–19]. Further, SF may even be cardiometabolically protective in overweight and obesity [14,25,26] due to its “expandability” and thus protection from the cardiometabolically adverse accumulation of VF [27,28]. 

 Given the clear limitation of currently used clinical measures of adiposity (BMI and waist circumference) to predict VF, development of cost-effective imaging techniques to quantify VF that would be suitable for everyday clinical practice and/or identification of robust circulating biomarkers of VF accumulation are warranted. 

## Supporting Information

File S1 **Supporting table and figures.** **Table S1**, Basic characteristics and adiposity measures for early and late puberty males and females. **Figure S1**, BMI and waist circumference as predictors of VF and SF quantities (VF and SF not adjusted for each other). **Figure S2**, suprailiac skinfold thickness and waist–to-hip ratio as predictors of VF and SF quantities (VF and SF not adjusted for each other). **Figure S3**, suprailiac skinfold thickness and waist-to-hip ratio as predictors of VF and SF quantities (VF and SF adjusted for each other).
(DOC)
