# Supplementary material for: Routine Clinical Measures of Adiposity as Predictors of Visceral Fat in Adolescence: A Population-Based Magnetic Resonance Imaging Study
Source: PLoS One. 2013 Nov 11;8(11):e79896. doi: 10.1371/journal.pone.0079896 (PMC3823587; doi:10.1371/journal.pone.0079896)

**Supplementary Tables**

***Table S1. Basic characteristics and adiposity measures for early and late puberty males and females***

|  | Males | | | | Females | | | |  | Sex Differences: p-value | |
| --- | --- | --- | --- | --- | --- | --- | --- | --- | --- | --- | --- |
| Variables | | Early Puberty  Mean ±SD | Late Puberty  Mean ±SD | p-value | | Early Puberty  Mean ±SD | Late Puberty  Mean ±SD | p-value | Early Puberty | | Late Puberty |
| Number | | 254 | 226 |  | | 83 | 436 |  |  | |  |
| Age (months) | | 168 ±17 | 192±19 | <0.0001 | | 156±11 | 186±21 | <0.0001 | <0.0001 | | 0.0007 |
| Height (cm) | | 162 ±10 | 173±7.3 | <0.0001 | | 153±6.8 | 161±6.0 | <0.0001 | <0.0001 | | <0.0001 |
| Puberty stage | |  | | | | | | | | | |
| Stage 1 | | 2% | - | - | | 1% | - | - | - | | - |
| Stage 2 | | 13% | - | - | | 1% | - | - | - | | - |
| Stage 3 | | 38% | - | - | | 14% | - | - | - | | - |
| Stage 4 | | - | 41% | - | | - | 57% | - | - | | - |
| Stage 5 | | - | 6% | - | | - | 27% | - | - | | - |
| BMI | |  |  |  | |  |  |  |  | |  |
| kg/m^2^ | | 20.9 ±4.3 | 22.5 ±4.2 | <0.0001 | | 18.9 ±3.5 | 22.2 ±3.9 | <0.0001 | 0.0001 | | 0.85 |
| log kg/m^2^ | | 1.31 ±0.08 | 1.35 ±0.08 | <0.0001 | | 1.27 ±0.07 | 1.34 ±0.07 | <0.0001 | <0.0001 | | 0.50 |
| percentile | | 58.0 ±29.4 | 59.5 ±28.2 | 0.58 | | 43.3 ±26.6 | 58.3 ±26.9 | <0.0001 | 0.0002 | | 0.58 |
| Waist | |  |  |  | |  |  |  |  | |  |
| cm | | 73.2 ±11 | 77.3 ±9.0 | <0.0001 | | 65.6 ±7.6 | 72.0 ±8.7 | <0.0001 | <0.0001 | | <0.0001 |
| log cm | | 1.86 ±0.06 | 1.89 ±0.05 | <0.0001 | | 1.81 ±0.05 | 1.85 ±0.05 | <0.0001 | <0.0001 | | <0.0001 |
| Hip | |  |  |  | |  |  |  |  | |  |
| cm | | 86.1 ±10 | 92.1 ±9.5 | <0.0001 | | 80.9 ±8.4 | 91.6 ±9.3 | <0.0001 | <0.0001 | | 0.68 |
| log (cm) | | 1.93 ±0.05 | 1.96 ±0.04 | <0.0001 | | 1.91 ±0.04 | 1.96 ± 0.04 | <0.0001 | <0.0001 | | 0.67 |
| Suprailiac skinfold | | | | | | | | | | | |
| mm | | 16.8 ±12 | 16.8±11 | 0.99 | | 16.1±11 | 21.4±10 | <0.0001 | 0.64 | | <0.0001 |
| log mm | | 1.13 ±0.29 | 1.14±0.27 | 0.66 | | 1.13±0.25 | 1.29±0.20 | <0.0001 | 0.90 | | <0.0001 |
| Waist/Hip | |  |  |  | |  |  |  |  | |  |
| cm/cm | | 0.850 ±0.059 | 0.845 ±0.054 | 0.36 | | 0.818 ±0.060 | 0.786 ±0.061 | <0.0001 | <0.0001 | | <0.0001 |
| log cm/cm | | -0.072 ±0.029 | -0.074 ±0.027 | 0.39 | | -0.088 ±0.031 | -0.106 ±0.033 | <0.0001 | <0.0001 | | <0.0001 |
| Visceral fat | |  |  |  | |  |  |  |  | |  |
| cm^2^ | | 228 ±226 | 233 ±225 | 0.81 | | 172 ±150 | 216 ±140 | 0.009 | 0.03 | | 0.22 |
| log cm^2^ | | 2.21 ±0.35 | 2.23 ±0.36 | 0.52 | | 2.14 ±0.26 | 2.26 ±0.24 | <0.0001 | 0.09 | | 0.12 |
| Subcutaneous fat | | | | | | | | | | | |
| cm^2^ | | 1070 ±1044 | 1045 ±1015 | 0.79 | | 933 ±809 | 1548 ±960 | <0.0001 | 0.27 | | <0.0001 |
| log cm^2^ | | 2.86 ±0.38 | 2.86 ±0.35 | 0.91 | | 2.87 ±0.28 | 3.12 ±0.25 | <0.0001 | 0.90 | | <0.0001 |

**SUPPLEMENTARY FIGURE Legends**

***Figure S1. BMI and waist circumference as predictors of VF and SF quantities (VF and SF not adjusted for each other)***

Multivariate linear regression models examining the relationships of BMI and waist circumference with each VF and SF (while not adjusting for each other) are shown in adolescent males and females. All relationships were also adjusted for potentially confounding effects of age and height when appropriate.

***Figure S2. Suprailiac skinfold thickness and waist–to-hip ratio as predictors of VF and SF quantities (VF and SF not adjusted for each other)***

Multivariate linear regression models examining the relationships of suprailiac skinfold thickness and waist/hip with each VF and SF are shown in adolescent males and females. All relationships were also adjusted for potentially confounding effects of age.

***Figure S3. Suprailiac skinfold thickness and waist-to-hip ratio as predictors of VF and SF quantities (VF and SF adjusted for each other)***

Multivariate linear regression models examining the relationships of suprailiac skinfold thickness and waist/hip with each VF and SF (while not adjusting for each other) are shown in adolescent males and females. All relationships were also adjusted for potentially confounding effects of age.


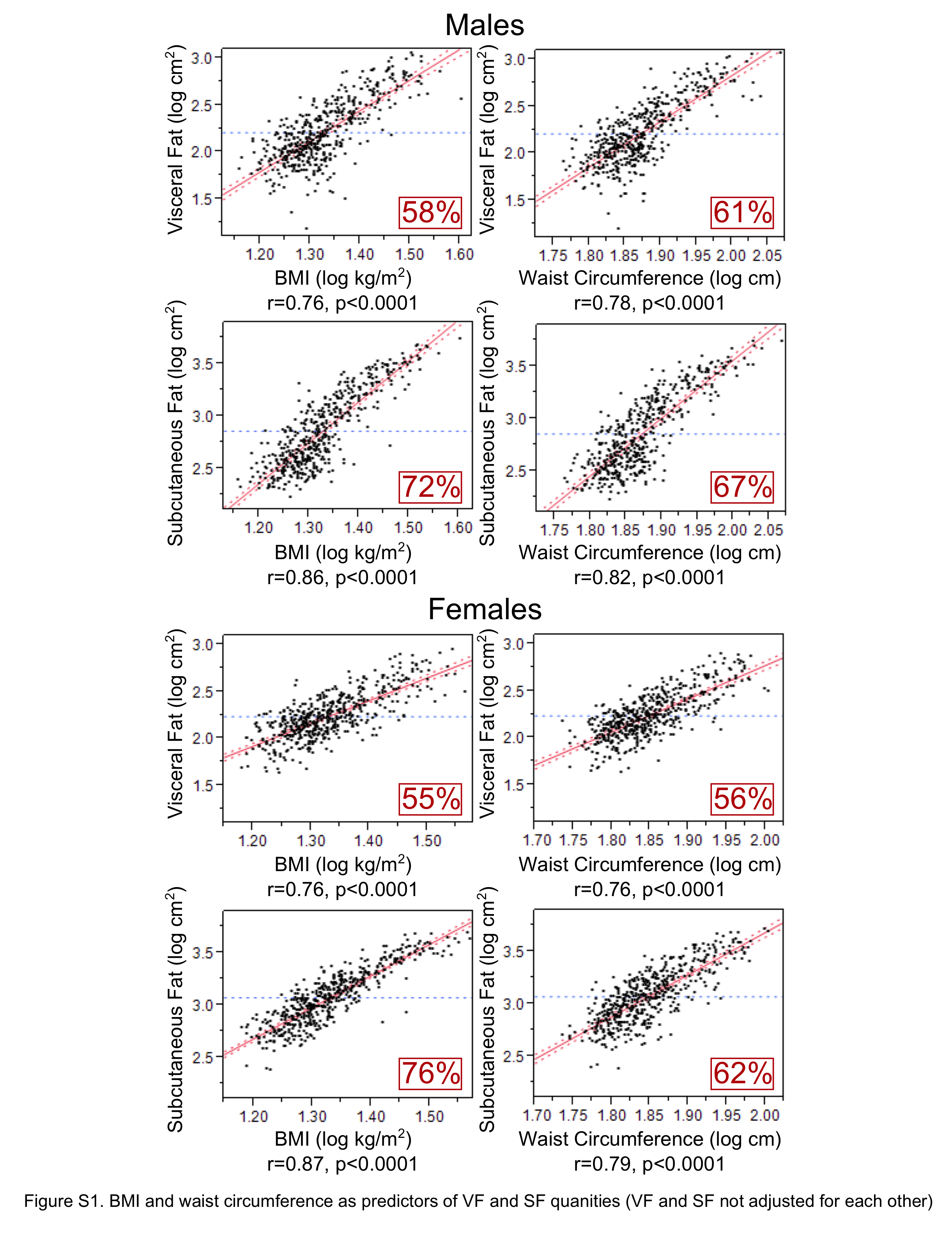


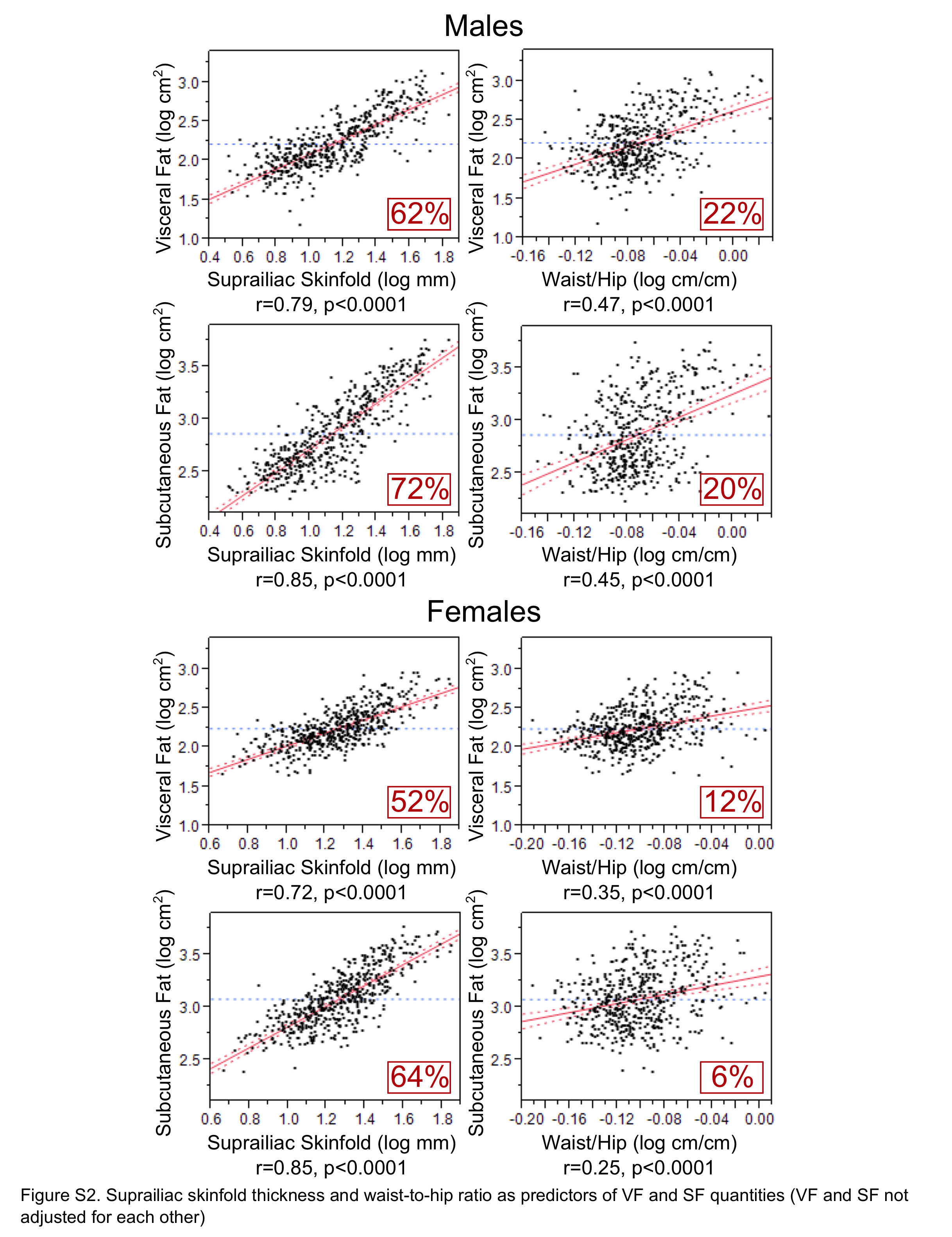


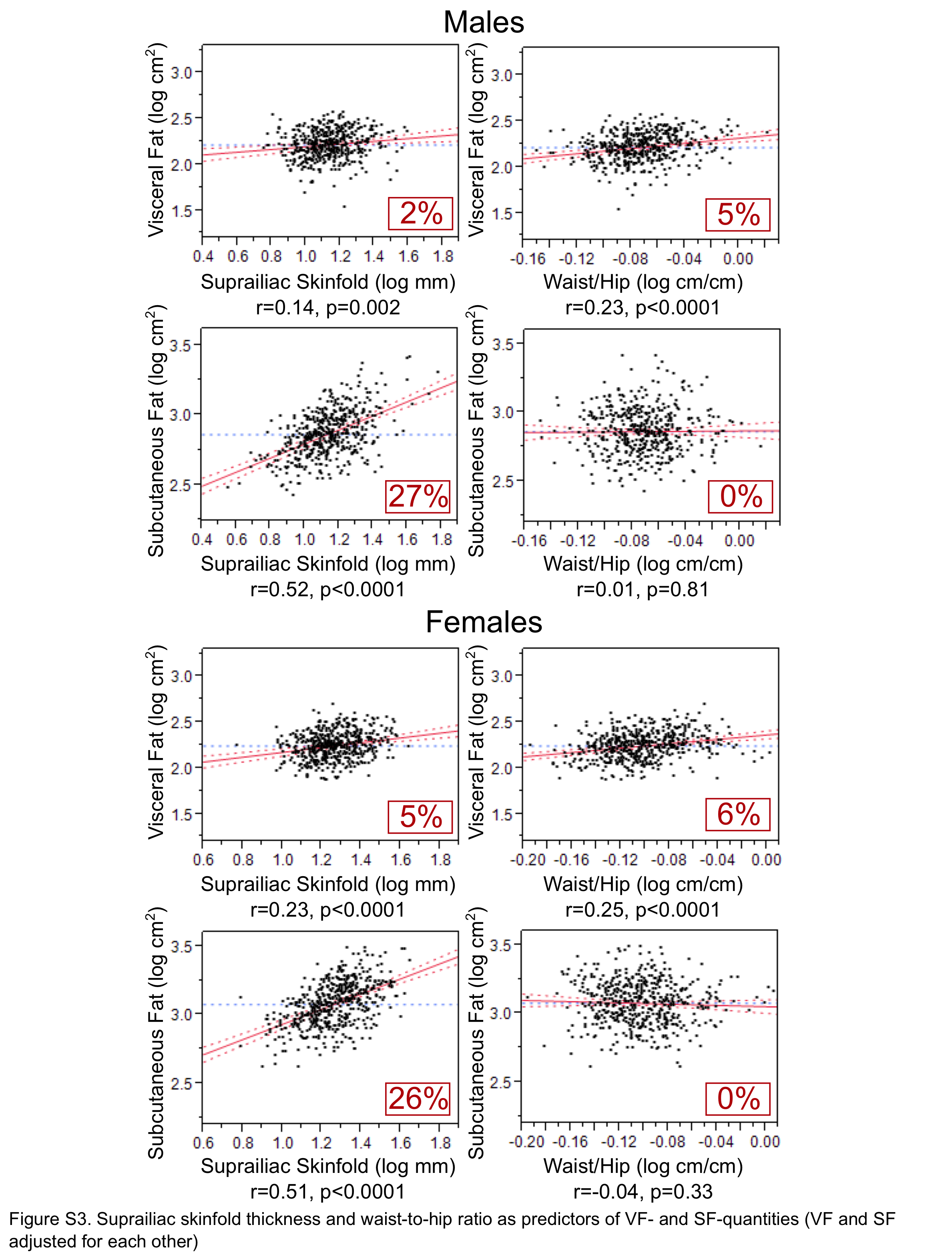

Supplement: File S1 — Supporting table and figures. Table S1, Basic characteristics and adiposity measures for early and late puberty males and females. Figure S1, BMI and waist circumference as predictors of VF and SF quantities (VF and SF not adjusted for each other). Figure S2, suprailiac skinfold thickness and waist–to-hip ratio as predictors of VF and SF quantities (VF and SF not adjusted for each other). Figure S3, suprailiac skinfold thickness and waist-to-hip ratio as predictors of VF and SF quantities (VF and SF adjusted for each other). (DOC) [file pone.0079896.s001.doc]
